# An artificial intelligence tool to predict fluid requirement in the intensive care unit: a proof-of-concept study

**DOI:** 10.1186/cc7140

**Published:** 2008-12-01

**Authors:** Leo Anthony Celi, L Christian Hinske, Gil Alterovitz, Peter Szolovits

**Affiliations:** 1 Laboratory of Computer Science, Massachusetts General Hospital, 50 Staniford Street, 7^th ^floor, Boston, MA 02114, USA; 2 Decision Systems Group, 900 Commonwealth Avenue, 3rd Floor, Boston, MA 02215, USA; 3 Children's Hospital Informatics Program, Enders Building 6th Floor, room 624.1, 320 Longwood Avenue, Boston, MA 02115, USA; 4 The Stata Center, Building 32, 32 Vassar Street, Cambridge, MA 02139, USA

## Abstract

**Introduction:**

The goal of personalised medicine in the intensive care unit (ICU) is to predict which diagnostic tests, monitoring interventions and treatments translate to improved outcomes given the variation between patients. Unfortunately, processes such as gene transcription and drug metabolism are dynamic in the critically ill; that is, information obtained during static non-diseased conditions may have limited applicability. We propose an alternative way of personalising medicine in the ICU on a real-time basis using information derived from the application of artificial intelligence on a high-resolution database. Calculation of maintenance fluid requirement at the height of systemic inflammatory response was selected to investigate the feasibility of this approach.

**Methods:**

The Multi-parameter Intelligent Monitoring for Intensive Care II (MIMIC II) is a database of patients admitted to the Beth Israel Deaconess Medical Center ICU in Boston. Patients who were on vasopressors for more than six hours during the first 24 hours of admission were identified from the database. Demographic and physiological variables that might affect fluid requirement or reflect the intravascular volume during the first 24 hours in the ICU were extracted from the database. The outcome to be predicted is the total amount of fluid given during the second 24 hours in the ICU, including all the fluid boluses administered.

**Results:**

We represented the variables by learning a Bayesian network from the underlying data. Using 10-fold cross-validation repeated 100 times, the accuracy of the model in predicting the outcome is 77.8%. The network generated has a threshold Bayes factor of seven representing the posterior probability of the model given the observed data. This Bayes factor translates into p < 0.05 assuming a Gaussian distribution of the variables.

**Conclusions:**

Based on the model, the probability that a patient would require a certain range of fluid on day two can be predicted. In the presence of a larger database, analysis may be limited to patients with identical clinical presentation, demographic factors, co-morbidities, current physiological data and those who did not develop complications as a result of fluid administration. By better predicting maintenance fluid requirements based on the previous day's physiological variables, one might be able to prevent hypotensive episodes requiring fluid boluses during the course of the following day.

## Introduction

The gold standard in evidence-based medicine is a well-designed, well-executed multi-centre prospective randomised controlled trial. However, in the intensive care unit (ICU), it would be impossible to perform such a study to determine whether every diagnostic test, monitoring device or treatment intervention leads to improved patient outcomes. Even when such trials are performed and subsequently published, they rarely, if ever, provide clear evidence on which to base the management of an individual patient.

Patients enrolled in these studies are heterogeneous, and conclusions are valid for the 'average' patient. Unfortunately, each patient is unique in terms of how he responds to an intervention. He may not benefit or, worse, may be harmed by a medication, device or procedure that has been shown to correlate with a good patient outcome 'on average'. In addition, these studies investigate one treatment at a time. In reality, treatments are given simultaneously to a patient in the ICU and interact with each other. The nature of these interactions is likely to vary from patient to patient, and perhaps even within the same patient at different points in time.

Over the years, we have adopted a multitude of diagnostic tests, monitoring devices and treatments in the ICU based on underpowered studies, in most cases non-randomised, which demonstrate modest benefits on soft clinical endpoints or intermediate outcomes. It is unclear which of these interventions contribute to survival benefit. Despite all the medical advances available in the ICU, less than half of patients who experience severe sepsis are alive one year later [1]. Another study found that mortality of pneumococcal bacteraemia has not changed over the past 50 years [2]. Finally, acute renal failure treated in the ICU with renal support therapy still carries a mortality of 64% to 79%, which has not significantly changed over the decades [3].

Over the past decade, we have witnessed the electronification of health care delivery and with it, the creation of large ICU databases of tremendous granularity and resolution. At the same time, the concept of personalised medicine emerged. The goal of personalised medicine is to provide the right treatment to the right patient at the right time. It involves the integration of genomics, proteomics, metabolomics, systems biology, bioimaging and other disciplines in order to characterise the uniqueness of a patient and predict his risk of developing a disease or his response to treatment. It is a tool that can potentially optimise care customisation in the ICU where it is needed the most, given how sick the patients are and how some treatments can lead to worse clinical outcomes. Unfortunately, the dynamic cytokine and neurohormonal milieu of the critically ill patient alters such processes as gene transcription and drug metabolism, rendering information derived during static non-diseased conditions of limited use. In this paper, we propose an alternative way of personalising medicine in the ICU using empiric data to build patient-specific and clinical scenario-specific models. We chose the prediction of fluid requirement of the critically-ill patient at the height of inflammatory response to explore the feasibility of this approach.

The first 72 hours after admission are critical for ICU patients. Whether the patient is being admitted for sepsis, acute coronary syndrome, multiple traumatic injuries, intracranial haemorrhage, burns or post-operative care after open heart surgery or organ transplantation, this period is characterised by systemic inflammatory responses fueled by a cytokine storm and the patient is most vulnerable to episodes of hypotension and consequently reduced organ perfusion. Suboptimal fluid management during this critical period leads to the release of more inflammatory cytokines and catecholamines that further worsen the haemodynamic status of the patient. As shown in a number of clinical studies, reduced tissue perfusion resulting from fluid under-resuscitation translates into increased illness severity and a longer ICU stay [4,5]. In practice, clinicians estimate the rate of maintenance fluids (usually in the range of 1 to 3 ml/kg/hour) by estimating fluid loss, a task that is very difficult in a critically ill patient because of the absence of a defined set of rules and guidelines for specific patient subsets in various clinical scenarios. In this study, we set out to see if we could predict the total amount of fluid administered to a patient on day two in the ICU, given the physiological data from the previous 24 hours.

## Materials and methods

The Laboratory of Computational Physiology at Massachusetts Institute of Technology developed and maintains the Multi-parameter Intelligent Monitoring for Intensive Care (MIMIC II) database, a high-resolution database of ICU patients admitted to the Beth Israel Deaconess Medical Center in Boston since 2003, who have been de-identified by removal of all protected health information. An Institutional Review Board approval was obtained from both Massachusetts Institute of Technology and Beth Israel Deaconess Medical Center for the development, maintenance and public use of a de-identified ICU database.

The MIMIC II database currently consists of data from more than 18,000 patients that have been de-identified and formatted to facilitate data-mining. The three sources of data are waveform data collected from the bedside monitors, hospital information systems and other third-party clinical information systems.

Using the MIMIC II database, we identified patients who were on vasopressor agents for more than six hours during the first 24 hours of their ICU admission. For each patient, we obtained demographic data and physiological variables during the first 24-hour period in the ICU. These variables included vital signs, those that affect and/or represent total body water, and those that reflect severity of illness (Table 1). Rather than representing one state of each variable, which is typically the worst value in severity scoring systems, we decided to include the following for each variable that we evaluated: mean, variance, maximum value, minimum value, number of measurements obtained and the last measurement taken during the first 24 hours, as this reflects whether the patient is improving, stablising or worsening compared with the worst value. Filtering was performed by deleting values that were outside the physiologically feasible range.

**Table 1:** Patient variables evaluated as possible predictors of maintenance fluid requirement.

**General demographics**	Age
	Sex
	Weight
	
**Physiological variables**	Blood pressure
	Heart rate
	
**Variables that affect and/or represent total body water**	Total fluid input during the first 24 hours
	Total fluid output during the first 24 hours
	Serum creatinine, as a surrogate marker of kidney function
	Serum sodium
	
**Variables that affect insensible fluid loss**	Body surface area
	Temperature
	
**Variables that reflect severity of illness**	Serum albumin
	Serum lactate
	Maximum number of vasopressors and inotropes
	Maximum number of sedatives and narcotic agents
	Serum bilirubin
	Haemoglobin
	Platelet count
	PaO_2_:FiO_2 _ratio

FiO_2 _= fraction of inspired oxygen; PaO_2 _= partial pressure of oxygen in arterial blood.

Using R software (R version 2.7.2, The R Foundation for Statistical Computing, Auckland, New Zealand), linear regression was performed using stepwise forward variable selection and with a 2:1 split sample approach (2/3 training data, 1/3 validation data). The total fluid administered during the second 24-hour period in the ICU was selected as the outcome variable. Using Bayesware Discoverer (Bayesware Discoverer Version 1.0, Massachusetts Institute of Technology, Cambridge, MA, USA), we also constructed a Bayesian network using the variables identified and extracted from the MIMIC II database. A Bayesian network is generated depicting the relation between variables based on the joint conditional probability distributions of the variables from the data set. The output is a graphical model representing the variables and their probabilistic dependencies. For our model generation, a maximum number of allowable parents was set at 10, and the threshold Bayes factor was set at 7. The variables, including the outcome variable, were divided at quartiles according to frequency.

Finally, the search order was arranged so that the outcome variable we are interested in, that is the total fluid intake for the second 24 hours after ICU admission, would have the largest number of nodes considered as potential parents. The accuracy of the model in predicting the outcome is calculated using 10-fold cross validation repeated 100 times.

## Results

There were a total of 3014 patients who were on at least one vasopressor agent for a minimum period of six hours during their first 24 hours in the ICU and whose total fluid intake and output were recorded.

**Figure 1:**
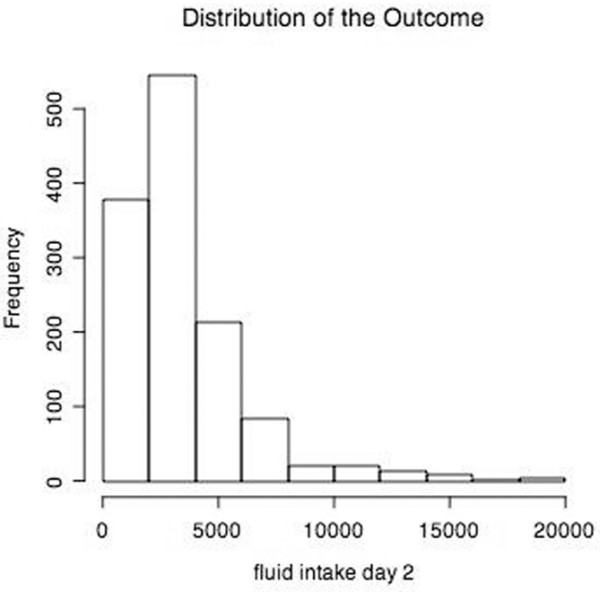
Distribution of total fluid intake on day two.

The distribution of the total fluid intake during the second day in the ICU, the outcome variable, was skewed towards the lower values (Figure 1). The values were therefore log transformed to approximate a more normal distribution for the linear regression model.

Using a stepwise forward variable selection on a 2:1 split sample approach, 14 variables were found to be predictive of the total amount of fluid given to the patient on day two in the ICU. The coefficients of the variables in the fitted model, their standard errors and the corresponding p values are shown in Table 2.

**Table 2:** Coefficients of the fitted linear regression model.

Variables	Estimate	Standard error	p value
Number of vasopressors	5.35e+02	2.38e+03	3.25e-06
Maximum heart rate (beats per minute)	1.12e+01	3.65	6.43e-09
Maximum haemoglobin (mg/L)	5.71e+01	8.76e+01	0.0021
Minimum haemoglobin (mg/L)	-5.17e+02	9.36e+01	1.03e-10
Variance of haemoglobin (mg/L)	-1.92e+02	4.72e+01	4.01e-08
Total fluid intake on day 1 (ml)	1.03e-01	2.14e-02	5.09e-05
Total fluid output on day 1 (ml)	-1.99e-01	3.68e-02	1.50e-06
Most recent platelet count (×10^9^/L)	1.19e+01	2.99	7.90e-08
Number of sedatives	2.74e+02	1.07e+02	7.73e-05
Age (years)	-1.15e+01	4.68	0.0106
Mean platelet count (×10^9^/L)	1.37e+01	2.97	4.17e-06
Minimum serum sodium (mEq/L)	1.68e+02	4.21e+01	7.27e-05
Most recent serum sodium (mEq/L)	8.33e+01	3.61+01	0.0212
Mean serum sodium (mEq/L)	1.67e+02	6.29e+01	0.0080

R-squared was calculated as a measure of the explained variation accounted for by the linear regression model. It is given by the formula:

R^2 ^= 1 - (SS_err_/SS_tot_)

where SS_err _is the residual sum of squares and SS_tot _is the sum of squares differences from the mean proportional to the variance. The adjusted R-squared value calculated was 0.25, suggesting that very little of the observed variation can actually be explained by the model. The linear regression model suffered from large variation with a high standard residual error, making it suboptimal for clinical application. For this reason, we shifted to a Bayesian network model to represent our variables and outcome.

**Figure 2:**
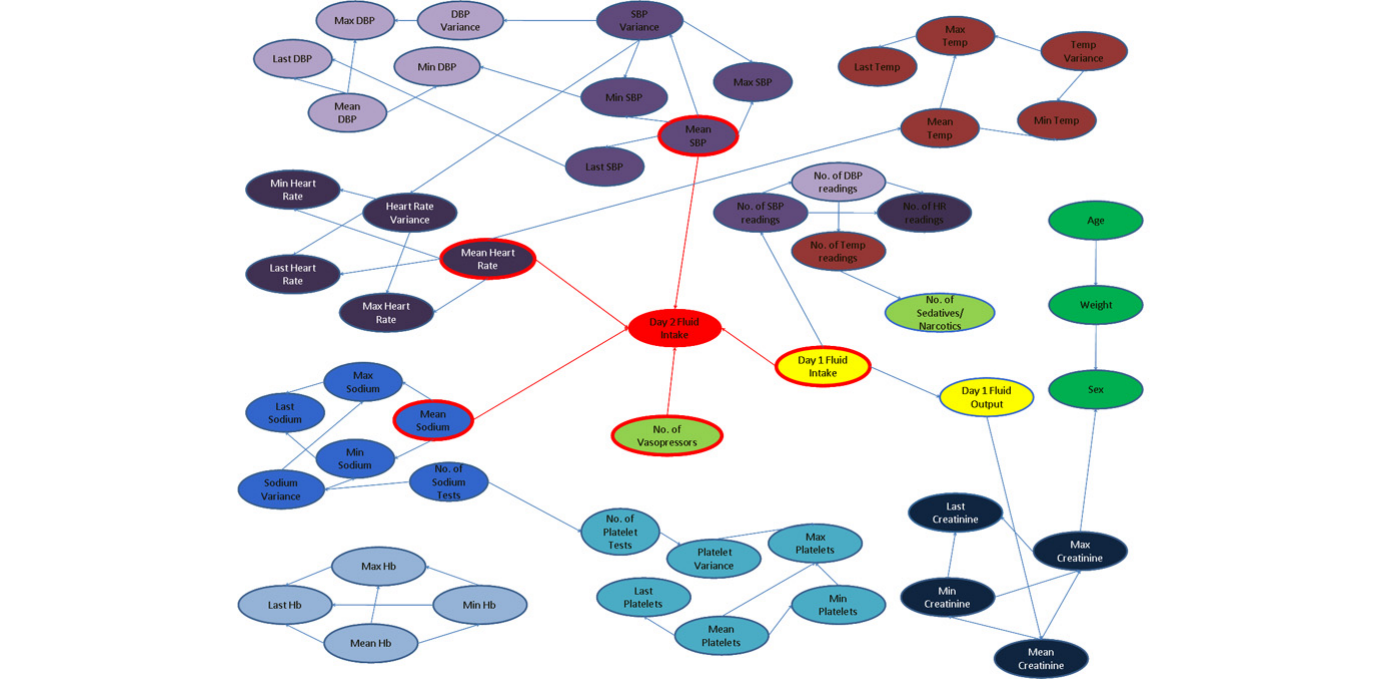
**Bayesian network model predicting maintenance fluid requirement on day two in the ICU**. DBP = diastolic blood presure; Hb = haemoglobin; Max = maximum; Min = minimum; SBP = systolic blood pressure; Temp = temperature.

Figure 2 illustrates the Bayesian network model generated from the MIMIC II database. For this particular data set, five variables were found to be correlated with the total fluid intake for the second 24 hours in the ICU: total fluid intake for the first 24 hours, number of vasopressor agents, mean systolic pressure, mean heart rate and mean serum sodium. Based on the model, the probability that a patient will require a certain range of fluid on day two can be predicted given the values of the variables that are direct parents of our outcome variable. The accuracy of the model in predicting the outcome variable was found to 77.8% on 10-fold cross validation repeated 100 times. This means the model generated from the training set, when applied to the validation set, was able to accurately predict the quartile of the total fluid administered on day two in 77.8% of cases.

The threshold Bayes factor is the smallest amount of evidence that can be claimed for the null hypothesis (no correlation between variables) or the strongest evidence against it on the basis of the observed data. This is the benchmark to compare it against a p value. The simplest relation between p values and Bayes factors are based on a Gaussian approximation. In that situation, the Bayes factor is calculated with the same numbers used to calculate a p value [6]. The formula is as follows: [7]

Bayes factor = *e*^-Z^2/2^

where Z is the number of standard errors from the null effect. This formula allows us to establish an exchange rate between the Bayes factor and p values in a Gaussian case. For a threshold Bayes factor of seven which was used to generate our model, the corresponding maximum p value is 0.05, assuming Gaussian distribution of the variables. Each link between two nodes in the network has a corresponding Bayes factor. These were not shown because prediction of a variable based on the values of the variables it is related to rests solely on the joint conditional probability distribution and not on the individual Bayes factors.

## Discussion

Figuring out the fluid requirement to maintain an adequate intravascular volume (and optimal preload) is difficult at the time of critical illness. In practice, clinicians fear over-estimating this fluid requirement. This may contribute to the occurrence of hypotensive episodes especially during the period of maximal systemic inflammatory response. These hypotensive episodes may be averted by being able to predict more accurately the fluid requirement of the patient as the disease process evolves in response to treatment or as a result of healing.

The goal of this proof-of-concept study is to explore the feasibility of supplementing traditional evidence-based medicine, expert opinion and clinical intuition with information from empiric data. A Bayesian network was generated between physiological variables obtained during the first 24 hours in the ICU and the total amount of fluid given on the second day in the ICU (maintenance fluid plus all the boluses the patients received) from a large database. A greedy search algorithm was used with the outcome variable of interest being evaluated first for potential parent nodes, and the demographic variables (age, sex and weight) being evaluated last. Given the values of the physiological variables from day one, the range of the total fluid given to the patient on day two can be predicted. Cross-validation was used to determine how well a Bayesian network model represents our data. In cross-validation, the model generated from the training set is evaluated against a previously unseen data. The accuracy of the model in predicting the outcome was 77.8%.

We suspect the reason why the accuracy of the Bayesian network generated from the data is not better may relate to the limitations of our methodology. The subset of patients included in the analysis is likely to still represent a heterogeneous group given that the reason why the patient was on vasoactive drugs was not considered. The patients likewise probably represent a wide spectrum as regards the degree of inflammatory response, with possible inclusion of patients who have a minimal amount of inflammation but were put on vasopressors nonetheless. What we would like to do in the future, when we have a larger database, is to specify a more homogeneous group of patients in terms of demographic variables, co-morbidities and clinical scenario.

Another potential source of model inaccuracy in the ICU is data noise. This includes device-related artifacts (e.g. arterial blood pressure dampening), laboratory errors, missing data and erroneous transcription, to name just a few. Filtering during data pre-processing was performed to reduce, but not obliterate, the impact of noise. The choice of the threshold Bayes factor (the likelihood of the model with links between the parent nodes and their children as compared with a model where the variables are independent) is thus crucial in preventing over-fitting when the data is unavoidably noisy.

A point of contention is whether to include clinical outcomes (e.g. resolution of acidosis, discontinuation of vasoactive agents, ICU length of stay or mortality) in the generation of the model. We elected to exclude these variables from the model generation because there are other variables that affect these clinical outcomes apart from fluid management (e.g. choice of antibiotics and timeliness of surgery if required). For this proof-of-concept study, we took a simple approach and focused on predicting how much fluid is given to a patient depending on physiological data obtained during the previous 24 hours, regardless of clinical outcome.

A number of studies have looked at the application of artificial intelligence tools in the ICU. Barbini and colleagues [8] and Cevenini and colleagues [9] compared different models in predicting ICU morbidity after cardiac surgery and found the Bayesian and logistic regression models to be superior to artificial neural network, scoring systems and k-nearest neighbour in terms of discrimination, generalisation and calibration for this particular task. Bayesian network has also been used to predict prognosis of head injured patients in the ICU [10], mortality of patients readmitted to the ICU [11] and likelihood of ventilator-associated pneumonia [12] and other nosocomial infections [13].

**Figure 3:**
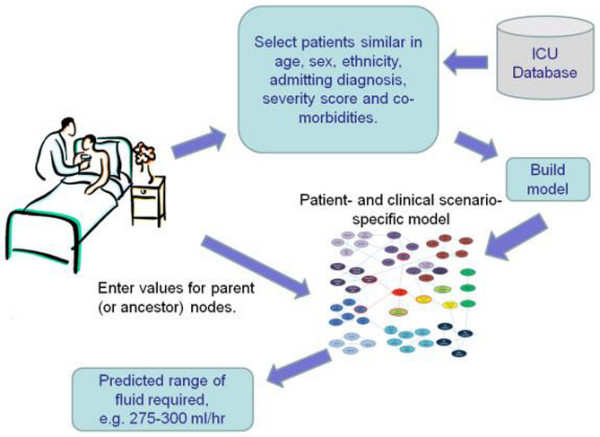
Artificial intelligence at the point-of-care in the ICU.

It is unlikely that we can replace clinician expertise with an intelligent software. We envision three important uses of artificial intelligence tools applied to empiric data. The first is to supplement clinical knowledge to support decisions in specialised, complicated problems where there may not be adequate evidence in the way of prospective randomised controlled trials. Figure 3 represents a diagram of how we envision the incorporation of this approach into clinical practice. The second is to potentially accelerate acquisition of clinical intuition by junior doctors in the ICU by 'learning' from their local database how senior intensivists managed identical patients in a specific clinical scenario. Finally, these tools might be of use for ongoing surveillance of medical devices, medications and interventions for clinical outcomes (rather than surrogate endpoints) especially in the ICU where these are sometimes adopted without clear evidence of long-term benefit.

We have taken a deductive approach in generating a model from empiric data. Combining such a deductive approach with an inductive knowledge base from domain expertise in pathological physiological processes and available ICU literature may provide a better tool in assisting clinicians in making decisions for individual patients in specific clinical scenarios.

## Conclusion

There are very few interventions performed in the ICU, whether for diagnostic, monitoring or treatment purposes, that are based on robust evidence. Even when prospective randomised controlled trials are available, they rarely, if ever, provide clear evidence on which to base the management of an individual patient. This project introduces the concept of using empiric data to obtain patient-specific and clinical scenario-specific recommendations in the ICU. Prediction of maintenance fluid is chosen as the problem domain to test the feasibility of the concept. A software application is envisioned that builds a model consisting of patients that are similar to an index patient in terms of age, gender, ethnicity, admitting diagnosis, severity score on admission and co-morbidities. Based on the model, physiological variables that are directly correlated with the outcome variable of interest are identified. The idea is to provide the values of these predictor variables from the index patient to the model, and a predicted range of fluid requirement is obtained from the joint conditional probabilities. We plan to evaluate the effect of the availability of this information in the ICU in an intention-to-treat prospective observational study. An adherence-to-protocol and on-treatment analyses will be incorporated into the design of the study.

## Key messages

• Only a handful of ICU interventions, whether for diagnostic, monitoring or treatment purposes, are based on well-designed, well-executed prospective randomised controlled trials.

• The project introduces the concept of mining empiric data using artificial intelligence to obtain patient-specific and clinical scenario-specific recommendations in the ICU.

• The idea is to build a model using patients that are similar to an index case with regards to age, sex, admitting diagnosis, co-morbidities and severity score.

• Prediction of maintenance fluid requirement in the ICU was chosen to explore the feasibility of the approach.

• The information obtained from the model may be useful in supplementing a clinician's knowledge base and intuition.

## Abbreviations

ICU: intensive care unit; MIMIC: Multi-parameter Intelligent Monitoring for Intensive Care.

## Competing interests

The authors declare that they have no competing interests.

## Authors' contributions

LC conceived of the study under the guidance of PS. LC and CH performed the data extraction and pre-processing, and with the help of GA, the data analysis. All authors read and approved the final manuscript.

## References

[B1] Yende S, Angus D (2007). Long-term outcomes from sepsis. Curr Infect Dis Rep.

[B2] Trampuz A, Widmer AF, Fluckiger U, Haenggi M, Frei R, Zimmerli W (2004). Changes in the epidemiology of pneumococcal bacteremia in a Swiss university hospital during a 15-year period, 1986–2000. Mayo Clin Proc.

[B3] Landoni G, Zangrillo A, Franco A, Aletti G, Roberti A, Calabrò MG, Slaviero G, Bignami E, Marino G (2006). Long-term outcome of patients who require renal replacement therapy after cardiac surgery. Eur J Anaesthesiol.

[B4] Rivers E, Nguyen B, Havstad S, Ressler J, Muzzin A, Knoblich B, Peterson E, Tomlanovich M, Early Goal-Directed Therapy Collaborative Group (2001). Early goal-directed therapy in the treatment of severe sepsis and septic shock. N Engl J Med.

[B5] Donati A, Loggi S, Preiser JC, Orsetti G, Münch C, Gabbanelli V, Pelaia P, Pietropaoli P (2007). Goal-directed intraoperative therapy reduces morbidity and length of hospital stay in high risk surgical patients. Chest.

[B6] Berger J (1985). Statistical Decision Theory and Bayesian Analysis.

[B7] Goodman SN (1999). Toward evidence-based medical statistics. 2: The Bayes factor. Ann Intern Med.

[B8] Barbini E, Cevenini G, Scolletta S, biagioli B, Giomarelli P, Barbini P (2007). A comparative analysis of predictive models of morbidity in intensive care unit after cardiac surgery – Part I: model planning. BMC Med Inform Decis Mak.

[B9] Cevenini G, Barbini E, Scolletta S, biagioli B, Giomarelli P, Barbini P (2007). A comparative analysis of predictive models of morbidity in intensive care unit after cardiac surgery – Part II: an illustrative example. BMC Med Inform Decis Mak.

[B10] Nikifordis G, Sakellaropoulos G (1998). Expert system support using Bayesian belief networks in the prognosis of head-injured patients of the ICU. Med Inform (Lond).

[B11] Ho K, Knuiman M (2008). Bayesian approach to predict hospital mortality of intensive care readmissions during the same hospitalisation. Anaesth Intensive Care.

[B12] Schurink CA, Visscher S, Lucas PJ, van Leeuwen HJ, Buskens E, Hoff RG, Hoepelman AI, Bonten MJ (2007). A Bayesian decision-support system for diagnosing ventilator-associated pneumonia. Intensive Care Med.

[B13] Schurink CA, Lucas PJ, Hoepelman IM, Bonten MJ (2005). Computer-assisted decision support for the diagnosis and treatment of infectious diseases in intensive care units. Lancet Infect Dis.

